# Sympathetic Ophthalmitis, and Certain Forms of Chronic Iritis

**Published:** 1860-09

**Authors:** E. L. Holmes

**Affiliations:** Chicago


### ARTICLE 2.

### SYMPATHETIC OPHTHALMITIS, AND CERTAIN FORMS OF CHRONIC IRITIS.

BY E. L. HOLMES, M. D., CHICAGO.

Much has been said and written regarding the ignorance of the profession in the Theory and Practice of Ophthalmic Medicine. That criticisms of this kind are just, may be inferred not only from the character of the authors who have alluded to the subject, but also from the acknowledgments of many individual members of the profession, and from the fact that the members of medical societies seldom take interest in discussions upon affections of the eye.

It is difficult to account for the general neglect of diseases so important, since there is scarcely any organ of sense, upon the health of which every one is so dependent as upon that of the eye, and there are few affections which require more care and judgment in their treatment than those of this delicate organ.

Diseases of the eye, especially of an inflammatory character, are very common in the Northwest, which is certainly a good reason why every physician engaged in country practice, should be able to treat them with skill. It has been said, and probably with truth, that it is more important for a physician in the country than for one in the city to understand the principles of surgery. We believe it still more important for him to possess an extensive knowledge of diseases of the eye and their treatment, since there are undoubtedly more cases in most of our communities in ophthalmic than in surgical practice, which require immediate and skillful treatment. We are confident that patients with diseases of the eye are in more danger of receiving injury from the treatment of uneducated practitioners than those suffering from injuries or surgical diseases. We believe more patients are rendered miserable and helpless by ignorance in treating diseases of the eye than in treating surgical cases.

This whole subject is one of great importance and worthy the serious consideration of every thoughtful physician. It is not our purpose, however, to dwell upon it at this time. We have alluded to it because the above remarks are, perhaps, especially applicable in reference to two classes of ophthalmic diseases, to which we desire to call the attention of those members of the profession who take comparatively little interest in ophthalmology. These classes of disease have of late been investigated with much care by eminent oculists, and facts have been established in regard to them, of which every practitioner should be well informed, since he may be thus able, in a few instances, perhaps, to prevent total loss of vision—a calamity which almost invariably renders the unfortunate sufferer dependent upon friends or the public.

The two classes of diseases to which we allude are, "Sympathetic inflammation of one eye, produced by certain injuries and inflammations of the other eye," and "Chronic iritis, especially that form of it accompanied by adhesions between the iris and lens."

### 1. *Sympathetic Ophthalmitis.*

During the past couple of years I have examined three cases in which, a few weeks after intra-ocular inflammation from injury had existed in one eye, the other was attacked with chronic inflammation of a similar nature, which had terminated, in spite of all treatment, with total loss of vision. At a late meeting of the Esculapian Society, held at Paris, Edgar county, I had an opportunity of listening to a verbal report of two such cases, which had fallen under the observation of Dr. S. York, some time after both eyes were seriously affected. Undoubtedly every experienced physician has either treated or observed patients who have become blind in consequence of an injury of one eye. It is unnecessary to report numerous cases, or to give a minute description of symptoms and course of the disease, since all this can be found in the works of Makenzie and other well known authors. To impress our readers, however, with an idea of the serious character of this disease, the following passages from Mackenzie may be quoted:

"Whenever I see sympathetic ophthalmitis, even in its first stage, I know that I have to contend with an affection, which, however slight its present symptoms may be, is one of the most dangerous inflammations to which the organ of vision is exposed." "I have very seldom seen an eye recover from sympathetic ophthalmitis."

It is difficult to explain why this disease, which is chiefly an inflammation of the iris and choroid, should be so much more obstinate than a similar inflammation under other circumstances.

The point of practical importance in the consideration of this subject is the treatment. Of the curative treatment of this disease I shall say a few words, in speaking of the second class of cases just mentioned.

The preventive treatment which has been found most beneficial by the most distinguished oculists of the present time, consists in the partial or total extirpation of the injured eye, *before the least symptom of inflammation has appeared in the other eye.* That this treatment is eminently successful may be inferred from the favorable manner in which it is mentioned by practical oculists. Almost every number of the London Ophthalmic Hospital Reports contains a history of several cases. We would also refer the reader to an article on the subject in the *New York Journal of Medicine*, for May, 1859, by F. J. Bunstead, M. D., Assistant Surgeon to the New York Eye Infirmary; and especially to a paper by v. Graefe, in the "Archiv fur Ophthalmologie." The operation, it must be remembered, as regards the sound eye, is preventive rather than curative.

Although the removal of the whole or a part of the eye is usually a simple operation, it is practically attended with certain difficulties. There is invariably in the mind of every patient a great horror at the thought of having an eye "cut out." A patient will often suffer long and excruciating pain, after vision in the injured eye has been irrecoverably lost, before he will submit to its removal. So long as the other eye remains unaffected he can scarcely be persuaded that it is in danger.

Another difficulty is found in the fact that many cases of mechanical injury of an eye, finally recover after months of painful inflammation, without unpleasant symptoms in the sound eye, and that it is, in fact, impossible to foretell in what cases the sympathetic inflammation will or will not make its appearance. This doubt, expressed by a surgeon, however intelligent, will often induce the patient to defer an operation so repugnant to his feelings.

In the paper of Graefe, to which I have alluded, three classes of cases are mentioned, as being especially liable to this form of disease.

1st. When a foreign body or a dislocated and enlarged lens is a source of irritation in one eye.

2d. When internal disorganization is progressing, with increase of pressure of the fluids of the eye outwards, rendering the walls of the globe more tense than natural.

3d. When, in commencing atrophy of one eye, (from chronic inflammations of iris and choroid,) there is tenderness on pressure over the region of the ciliary processes.

Even in these cases, it is true, the sound eye will sometimes escape sympathetic ophthalmitis. Still, with all this difficulty of prognosis and with the prejudices and fears of the patient we consider it as the duty of every surgeon in these cases where vision is lost, to advise either the total or partial extirpation of the eye, before the symptoms of disease appear in the other eye.

We believe it is better to pursue this course, even in a large number of cases where the sound eye might possibly have escaped, than to run the risk, in a single instance, of losing both eyes. In the cases of sympathetic ophthalmitis which have been reported, it has been found that the average length of time which elapses after the injury, before the other eye begins to suffer, is about five weeks; but the injured eye often remains for months affected with chronic ophthalmitis, afflicting the patient with severe pain, and unfitting him for the ordinary duties of life. An early operation would spare all this suffering, since the wound usually heals in about fifteen days.

I think any one who has had much experience in treating injuries of the eyes, must be fully convinced that many patients with penetrating wounds of the cornea and lens, attended with protrusion and inflammation of the iris and choroid, and with loss of vision, would escape much distress by resorting to the immediate removal of the anterior portion of the eye. Patients, however, will seldom submit to an operation till worn out with pain and loss of sleep, and threatened with loss of health.

The method of removing the eye, as now recommended by ophthalmic surgeons, is simple, and usually without danger. A circular incision is made through the conjunctiva, parallel to the cornea, and the recti muscles severed close to their sclerotical insertion, as in the operation for strabismus. The conjunctiva is then separated from the sclerotica and the optic nerve divided by a pair of blunt-pointed, curved scissors, passed behind the globe, when the eye can be readily removed from the orbit and the operation finished by cutting the oblique muscles. It is often advisable to enlarge the palpebral fissure by slitting the tissues at the external angle, especially when the eye is hypertrophied.

The operation, as thus performed, leaves the muscles and nearly the whole conjunctiva, which usually heal in about a fortnight, and form a "stump" sufficient for an artificial eye. In many instances the excision of the cornea is sufficient. It is not, however, reliable, for the inflamed choroid and adjacent membranes may continue a source of irritation which can only be relieved by a total extirpation of the globe. Moreover the operation is not unfrequently followed by serious hemorrhage, since the inflammation has caused an enlargement of the vessels of the retina and charoid. The walls of these vessels, especially of the retina, are exceedingly delicate and often rupture "spontaneously" when the pressure of the humors within the globe is removed, as in excising the cornea. The operation for the extirpation of the eye is almost entirely free from this objection, as it is seldom attended with much loss of blood.

(To be continued.)

